# Particulate matter air pollution and national and county life expectancy loss in the USA: A spatiotemporal analysis

**DOI:** 10.1371/journal.pmed.1002856

**Published:** 2019-07-23

**Authors:** James E. Bennett, Helen Tamura-Wicks, Robbie M. Parks, Richard T. Burnett, C. Arden Pope, Matthew J. Bechle, Julian D. Marshall, Goodarz Danaei, Majid Ezzati

**Affiliations:** 1 Department of Epidemiology and Biostatistics, School of Public Health, Imperial College London, London, United Kingdom; 2 MRC Centre for Environment and Health, Imperial College London, London, United Kingdom; 3 Population Studies Division, Health Canada, Ottawa, Ontario, Canada; 4 Department of Economics, Brigham Young University, Provo, Utah, United States of America; 5 Department of Civil & Environmental Engineering, University of Washington, Seattle, Washington, United States of America; 6 Harvard T. H. Chan School of Public Health, Boston, Massachusetts, United States of America; 7 WHO Collaborating Centre on NCD Surveillance and Epidemiology, Imperial College London, London, United Kingdom; Columbia University, UNITED STATES

## Abstract

**Background:**

Exposure to fine particulate matter pollution (PM_2.5_) is hazardous to health. Our aim was to directly estimate the health and longevity impacts of current PM_2.5_ concentrations and the benefits of reductions from 1999 to 2015, nationally and at county level, for the entire contemporary population of the contiguous United States.

**Methods and findings:**

We used vital registration and population data with information on sex, age, cause of death, and county of residence. We used four Bayesian spatiotemporal models, with different adjustments for other determinants of mortality, to directly estimate mortality and life expectancy loss due to current PM_2.5_ pollution and the benefits of reductions since 1999, nationally and by county. The covariates included in the adjusted models were per capita income; percentage of population whose family income is below the poverty threshold, who are of Black or African American race, who have graduated from high school, who live in urban areas, and who are unemployed; cumulative smoking; and mean temperature and relative humidity. In the main model, which adjusted for these covariates and for unobserved county characteristics through the use of county-specific random intercepts, PM_2.5_ pollution in excess of the lowest observed concentration (2.8 μg/m^3^) was responsible for an estimated 15,612 deaths (95% credible interval 13,248–17,945) in females and 14,757 deaths (12,617–16,919) in males. These deaths would lower national life expectancy by an estimated 0.15 years (0.13–0.17) for women and 0.13 years (0.11–0.15) for men. The life expectancy loss due to PM_2.5_ was largest around Los Angeles and in some southern states such as Arkansas, Oklahoma, and Alabama. At any PM_2.5_ concentration, life expectancy loss was, on average, larger in counties with lower income and higher poverty rate than in wealthier counties. Reductions in PM_2.5_ since 1999 have lowered mortality in all but 14 counties where PM_2.5_ increased slightly. The main limitation of our study, similar to other observational studies, is that it is not guaranteed for the observed associations to be causal. We did not have annual county-level data on other important determinants of mortality, such as healthcare access and quality and diet, but these factors were adjusted for with use of county-specific random intercepts.

**Conclusions:**

According to our estimates, recent reductions in particulate matter pollution in the USA have resulted in public health benefits. Nonetheless, we estimate that current concentrations are associated with mortality impacts and loss of life expectancy, with larger impacts in counties with lower income and higher poverty rate.

## Abstract 2

Majid Ezzati and colleagues study associations between particular matter air pollution and life expectancy at county level across the USA.

## Author summary

**Why was this study done?:**

- Exposure to fine particulate matter pollution (PM_2.5_) is hazardous to health.
- This study was done to estimate the longevity benefits of current air pollution regulations and of further lowering concentrations.

**What did the researchers do and find?:**

- We used vital registration and population data from the contiguous United States between 1999 and 2015.
- We used a statistical model, adjusted for other determinants of mortality such as poverty, to directly estimate mortality and life expectancy loss due to current PM_2.5_ pollution.
- PM_2.5_ pollution in excess of the lowest observed concentration (2.8 μg/m^3^) was responsible for an estimated 15,612 deaths (95% credible interval 13,248–17,945) in females and 14,757 deaths (12,617–16,919) in males.
- These deaths lowered national life expectancy by an estimated 0.15 years for women and 0.13 years for men.

**What do these findings mean?:**

- Current PM_2.5_ pollution continues to contribute to mortality and loss of longevity in the USA, with larger harms in poorer counties.
- Further lowering PM_2.5_ pollution is likely to benefit the health of the entire US population and lower health inequalities.

## Introduction

**Fig 1:**
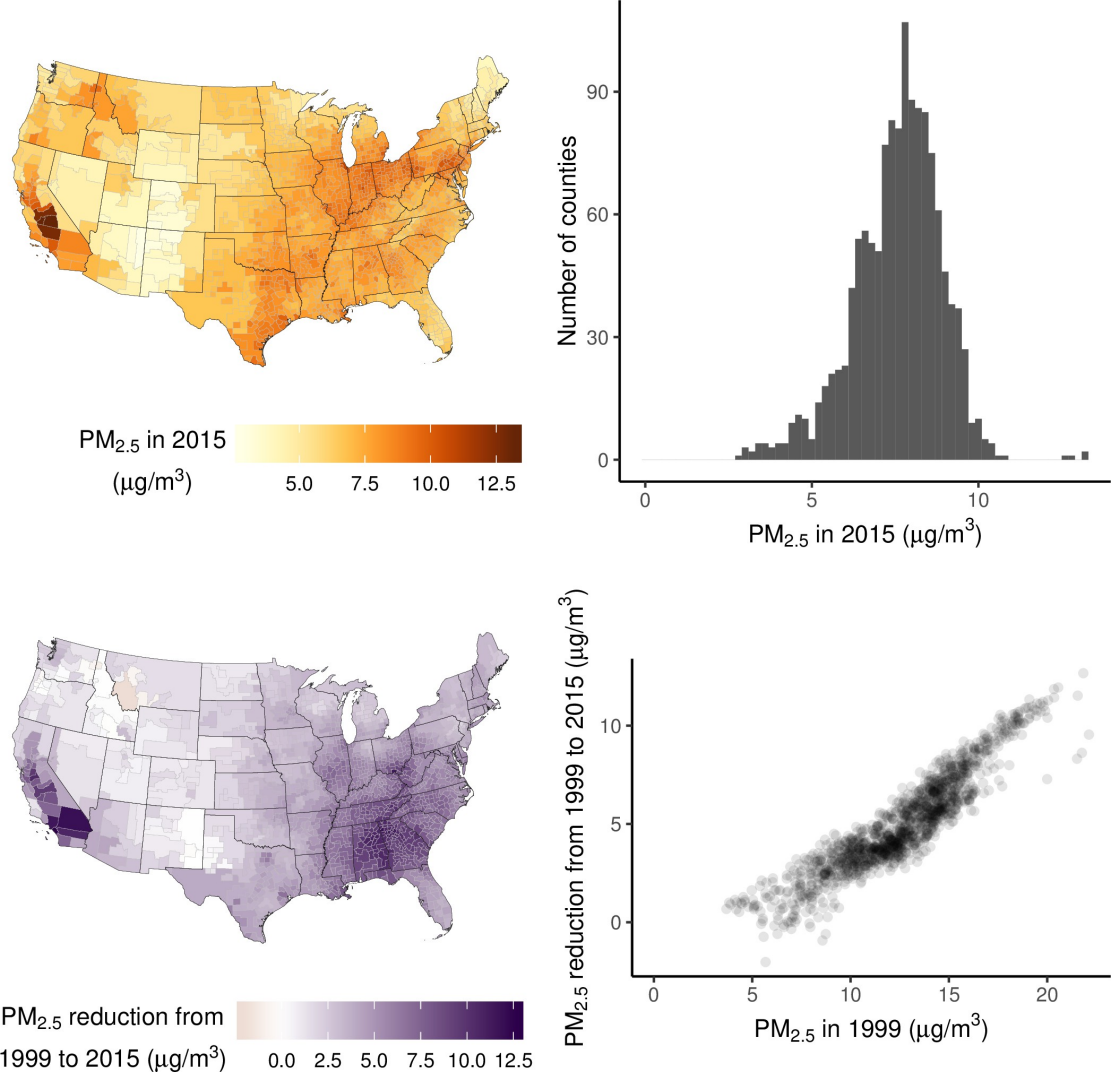
Population-weighted average PM_2.5_ concentration in 1,339 counties or merged county units (see Methods for description of analysis units). (A) Concentrations in 2015. (B) Distribution of concentrations in 2015. (C) Reductions in concentrations from 1999 to 2015. (D) Relationship between concentration reductions from 1999 to 2015 and 1999 concentrations. PM_2.5_ concentrations in merged county units are population-weighted averages of constituent counties. PM_2.5_, fine particulate matter.

Clean air policies and innovations in automotive, power generation, and industrial technologies have substantially improved air quality in the United States of America and other high-income countries [1]. These efforts were largely motivated by the evidence on the health hazards of air pollutants, especially fine particulate matter (PM_2.5_) [2–7]. Nonetheless, in 2015, nearly 9% of the US population lived in counties with PM_2.5_ concentrations above the WHO Air Quality Guideline of 10 μg/m^3^ [8], and another 89% in counties with concentrations of 5–10 μg/m^3^ (Fig 1). Recent epidemiological cohorts have observed adverse health impacts of PM_2.5_ even at these relatively low concentrations [6,9–12]. These findings indicate that further reducing PM_2.5_ is likely to lower mortality, especially from cardiorespiratory diseases, and increase longevity, as did the late 20th-century reductions [1]. Despite this evidence, there is not only resistance to more stringent control of PM_2.5_ but also attempts to roll back current standards [13–19].

Evidence-based air pollution regulations and policies require quantitative estimates of the health effects of air pollution in the low-concentration range for the contemporary US population, both in terms of deaths averted as a result of past reductions and those avertable if current concentrations are further reduced [20]. Previous estimates of the mortality burden of air pollution mostly extrapolated risk coefficients from epidemiological cohorts to the contemporary US population (see Methods) [21–24]. The only direct analyses of longevity loss due to air pollution focused on a subset of metropolitan areas [1,25], largely before concentrations were at their current relatively low levels, which are the subject of policy debate. Therefore, the fundamental questions of the benefits of current regulations and of further lowering concentrations for preventing deaths and increasing longevity have not been directly answered.

In this study, we used publicly available data to directly estimate the number of deaths, by age group and sex, and loss of life expectancy due to current PM_2.5_ concentrations in the entire contiguous USA (i.e., excluding Alaska and Hawaii), as well as the benefits of reductions since 1999. We present estimates of averted and avertable deaths and improved longevity nationally as well as at the small-area (county) level, which shows where the current death toll of air pollution is largest and where the health benefits of past controls have been greatest.

## Methods

### Study design

Our aim was to quantify, nationally as well as at the small-area (county) level, the current mortality burden of PM_2.5_ pollution and the averted mortality as a result of recent PM_2.5_ reductions. The most common source of information on the health effects of air pollution exposure is from prospective cohort studies [2–7,10,12]. By design, prospective cohort studies provide information on the risk associated with PM_2.5_ in a specific group of participants after a follow-up period, during which other determinants of population health also change. Therefore, estimates of excess mortality and loss of life expectancy due to air pollution in the contemporary population through application of risk estimates from prospective cohorts [6,21] implicitly assume that the magnitude of risk associated with PM_2.5_ in the prospective cohort participants is the same as the contemporary general population.

A direct approach for measuring the mortality burden of air pollution is to analyse the association of death rates with PM_2.5_ concentration across a large number of small-area units, such as counties, appropriately adjusting for other determinants of mortality [1,25–28]. Here, we used four Bayesian spatiotemporal models, with different degrees of adjustment for county characteristics that may also affect mortality, to directly estimate the mortality risk associated with PM_2.5_ in US counties from 1999 to 2015. We then used these associations to calculate excess deaths and life expectancy loss associated with current PM_2.5_ concentrations, as well as the benefits of reductions since 1999, for all counties in the contiguous USA. The study did not have a protocol or prespecified analysis plan.

We began the analysis in 1999 for two reasons: First, reliable data on PM_2.5_ throughout the USA became available in 1999, when the US Environmental Protection Agency (EPA) set up the Federal Method network of PM_2.5_ monitors [29]. Second, the USA switched from the ninth revision to the tenth revision of the International Classification of Disease (ICD) system in 1999 [30]. Therefore, the post-1999 data use a consistent system of the assignment of the medical cause of death.

### Data sources

We used data on deaths by sex, age, underlying cause of death, and county of residence from vital registration through the National Center for Health Statistics (NCHS) (https://www.cdc.gov/nchs/nvss/dvs_data_release.htm) and on population from the NCHS bridged-race data set (https://www.cdc.gov/nchs/nvss/bridged_race.htm) from 1999 to 2015. From 1999 to 2015, there were a total of 41.9 million deaths in the contiguous USA; 18.4 million of these deaths were from cardiorespiratory diseases (ICD-10 codes beginning with I and J), for which there is strong evidence of an association with air pollution.

Annual average PM_2.5_ concentrations were from an integrated geographic regression model detailed elsewhere [31]. In summary, the modelling of PM_2.5_ involved the application of a universal kriging approach to monitoring data and hundreds of geographic variables. The number of monitors that informed the estimates ranged from 599 in 1999 to 1,114 in 2002. There were more than 750 monitors for every year from 2000 to 2015. The variables in the model include population, elevation, vegetation, imperviousness, distance to traffic and other emission sources (for example, roads and intersections, railroad, airport, port, large point sources, etc.), and land use and cover (for example, forestland, rangeland, water, urban or built-up land divided into residential, commercial, etc.). The model also included satellite-derived estimates of particle pollution based on optical depth. The satellite-driven estimates help inform large-scale variability due to meteorological factors and regional sources, whereas the emissions and land use and land cover variables inform more localised concentration variability. Model performance was evaluated using cross-validation by leaving out some of the monitoring data from the model, which are then compared with model prediction. Annual PM_2.5_ concentrations were predicted for all years from 1999 to 2015 at all 2010 census block centroids in the contiguous USA and were aggregated by population-weighting to county level. The PM_2.5_ concentrations can be downloaded freely through https://www.caces.us/data.

We also gathered publicly available data on other county characteristics that may affect mortality:

- Per capita income ($), adjusted for inflation using the year 2000 as a baseline, from the CA1 data set from the Bureau of Economic Analysis (https://apps.bea.gov/regional/downloadzip.cfm) (used as log-transformed).
- Percentage of population whose family income is below the poverty threshold, from the US Census Bureau’s Small-Area Income and Poverty Estimates data set (https://www.census.gov/programs-surveys/saipe/data/datasets.All.html).
- Percentage of population who are of Black or African American race, through the NCHS bridged-race data set (https://www.cdc.gov/nchs/nvss/bridged_race.htm).
- Education, measured as percentage of population above the age of 25 years who have a high-school diploma or above, from the US Department of Agriculture (https://www.ers.usda.gov/data-products/county-level-data-sets/download-data/). The data set contained US Decennial Census data for 1970, 1980, 1990, and 2000 and American Community Survey 5-year estimates for the period 2011 to 2015. We linearly interpolated between 1990 and 2000 data to estimate the percentage of the population who had graduated from high school for 1999. We similarly estimated values for each county for years from 2001 to 2010 by linearly interpolating between 2000 and 2011 data.
- Unemployment, measured as percentage of population who are unemployed, calculated by dividing the number of people who are unemployed—available from the Bureau of Labor Statistics (https://www.bls.gov/lau/)—by total county population.
- Urbanicity, measured as percentage of population who live in urban areas, from the US Census Bureau’s decennial census data for 2000 and 2010 (https://factfinder.census.gov/faces/nav/jsf/pages/index.xhtml). We linearly interpolated between 2000 and 2010 data to estimate the percentage of population living in urban areas for 2001 to 2009. We used data from the year 2000 for 1999, and those from 2010 for 2011 to 2015.
- Cumulative smoking, proxied by log-transformed age-standardised lung cancer death rates as in previous population-based analyses [1,32–34]. We calculated lung cancer death rates using the abovementioned deaths on lung cancer (ICD 10 codes C33 and C34) and population. We used lung cancer death rates because when smoking prevalence or intensity change, it is a better measure of the mortality risk than current levels [33]. Further, there are data on lung cancer death rates, but not on smoking prevalence, for every county and analysis year.
- Temperature and relative humidity, from ERA-Interim (https://apps.ecmwf.int/datasets/data/interim-full-daily/levtype=sfc/), which combines predictions from a physical model with in situ and satellite measurements [35]. We used gridded 4-times-daily estimates at a resolution of 80 km to generate yearly temperature and relative humidity values by county throughout the analysis period.

### Units of analysis

In 2015, there were 3,108 counties in the contiguous USA. Over time, some counties were merged or split to create new counties. To avoid inconsistent analysis units over time, such counties were combined for the entire analysis period in order to have an unambiguously consistent set of analysis units. This resulted in 3,082 nonoverlapping county units in the contiguous USA with consistent boundaries for the entire analysis period.

The populations of these 3,082 counties from 1999 to 2015 ranged from 55 to 10,112,255. In small counties, some age groups had zero population in some years, which makes it impossible to calculate death rates. To avoid zero populations, we recursively merged counties with sex-specific populations smaller than 25,000 in any year with their neighbours until the populations in all sex-year combinations surpassed 25,000 in the newly formed county units. We merged counties without crossing state boundaries or combining urban and rural counties. We determined the urbanicity of each county from the NCHS’s Urban–Rural Classification Scheme for Counties in 2006 (https://www.cdc.gov/nchs/data_access/urban_rural.htm), which divides counties into six categories—large central metro, large fringe metro, medium metro, small metro, micropolitan, and noncore—based on their population and overlap with metropolitan or micropolitan statistical areas. We considered counties categorised under the first four as urban counties and the last two as rural counties. Using this algorithm, we created a total of 1,339 county units that covered the entire contiguous USA that were used for statistical analyses described below. PM_2.5_ concentrations for each of the constituent counties were population-weighted up to the level of the newly formed county units. The median absolute difference in PM_2.5_ concentration between the merged counties and their constituents from 1999 to 2015 was 0.28 μg/m^3^. The sociodemographic, environmental, and mortality characteristics of the merged counties are summarised in Table 1.

**Table 1:** Summary statistics by percentile of social, environmental, and epidemiological characteristics of the merged county units used in the analysis.

		1999					2015				
	Sex	1^st^	25^th^	50^th^	75^th^	99^th^	1^st^	25^th^	50^th^	75^th^	99^th^
**Population**	F	12,673	36,694	50,274	82,961	855,760	13,487	38,451	54,216	101,869	1,057,248
	M	12,991	35,560	48,802	79,392	844,233	13,409	38,328	53,837	99,255	1,055,120
**Cardiorespiratory as proportion of all deaths (%)**	F	41.1	49.2	51.8	54.5	62.1	31.8	38.9	41.3	44.4	51.8
	M	37.7	45.3	47.8	50.2	56.7	32.2	38.3	40.5	43.0	49.4
**Life expectancy at birth (years)**	F	75.4	78.1	79.4	80.4	83.0	75.4	78.7	80.3	81.9	85.8
	M	68.0	72.2	73.8	75.3	78.4	69.2	73.6	75.5	77.3	81.4
**Population who are of Black or African American race (%)**	F	0.1	0.8	3.6	14.1	57.0	0.6	1.6	4.7	14.9	57.8
	M	0.1	1.4	4.4	13.3	54.4	0.9	2.5	5.9	14.5	54.3
**Lung cancer death rate (age standardised, per 100,000)**	F	12.6	32.3	41.2	50.1	83.4	9.1	29.8	38.5	48.4	80.5
	M	25.0	67.6	87.4	109.6	162.5	14.4	43.3	60.3	77.6	129.5
**PM**_**2.5**_		5.0	10.4	12.7	14.6	19.7	3.6	6.8	7.7	8.5	10.1
**Temperature (annual mean, C)**		5.3	10.1	13.2	16.5	23.0	5.4	10.2	13.4	16.8	24.2
**Relative humidity (annual mean, %)**		41.5	67.1	69.5	71.9	77.3	45.8	66.9	69.5	71.8	77.7
**Income ($1,000s adjusted for inflation using the year 2000 as a baseline)**		16.5	21.7	24.0	27.7	47.1	20.4	25.7	28.7	32.7	60.1
**Poverty (% of population whose family income is below the poverty threshold)**		3.8	9.0	11.7	14.9	26.6	5.2	11.7	15.0	18.7	31.1
**Education (% of population above the age of 25 years who have a high-school diploma or above)**		57.9	72.9	79.8	83.8	91.9	70.6	82.9	87.5	90.4	95.3
**Unemployment (%)**		1.6	3.1	4.0	5.6	12.6	2.7	4.5	5.4	6.3	10.4
**Urbanicity (% of population who live in urban areas)**		6.8	37.7	56.8	77.8	99.9	7.6	38.9	60.9	80.3	99.8

**Abbreviations:** PM_2.5_, fine particulate matter.

### Statistical methods

We used four Bayesian spatiotemporal models, with different degrees of adjustment for county characteristics that may also affect mortality, to directly estimate the proportional (percent) increase in county-level age-specific death rates from cardiorespiratory diseases associated with the county’s annual PM_2.5_ concentration. The four models had similar structures but differed in the extent of adjustment for county characteristics that may also influence mortality, as described below and shown in Table 2. For a given sex and age group, the number of deaths in each county in each year follows a Poisson distribution:

$${deaths}_{county-year}∼Poisson({death\;rate}_{county-year}∙{population}_{county-year})$$

**Table 2:** Models used for relating county death rates to PM_2.5_ concentrations.

Model		Common terms		County terms		Pollution terms		Covariates		Overdispersion
Unadjusted	**log**(***death rates***_***county*−*year***_) =	***α***_**0**_**+*β***_**0**_**∙*year*+*ν***_***year***_			+	***γ*∙*PM***_***county−year***_			+	***ε***_***county−year***_
Covariate	**log**(***death rates***_***county*−*year***_) =	***α***_**0**_**+*β***_**0**_**∙*year*+*ν***_***year***_			+	***γ*∙*PM***_***county−year***_	+	$\sum_{i=1}^{i=9}\theta_{i}∙X_{i-county-year}$	+	***ε***_***county−year***_
Covariate-and-county	**log**(***death rates***_***county*−*year***_) =	***α***_**0**_**+*β***_**0**_**∙*year*+*ν***_***year***_	+	***α***_***ccounty***_	+	***γ*∙*PM***_***county−year***_	+	$\sum_{i=1}^{i=9}\theta_{i}∙X_{i-county-year}$	+	***ε***_***county−year***_
Restrictive	**log**(***death rates***_***county*−*year***_) =	***α***_**0**_**+*β***_**0**_**∙*year*+*ν***_***year***_	+	***α***_***ccounty***_+***β***_***ccounty***_∙***year***	+	***γ*∙*PM***_***county−year***_	+	$\sum_{i=1}^{i=9}\theta_{i}∙X_{i-county-year}$	+	***ε***_***county−year***_

**Abbreviations:** PM, particulate matter.

In all four models, log-transformed death rates were modelled as a sum of components that depend on time and air pollution, with some models also including terms for other time-varying county characteristics. In this specification, the coefficient of the PM_2.5_ term represents the logarithm of the proportional change in death rate for each unit additional PM_2.5_ concentration, akin to proportional hazard models used in analyses of prospective cohorts.

All models contained terms to capture the overall level and rate of change of mortality, with *α*_0_ as the common intercept for log-transformed death rates and *β*_0_ the common time slope. Nonlinear trends were captured by a first-order random walk *ν*_*year*_ [36]. All models also included a term that relates log-transformed death rate to county PM_2.5_ concentration, with the coefficient *γ* representing the logarithm of the rate ratio per 1 μg/m^3^ higher concentration. *ε*_*county−year*_ is an overdispersion term that captures the variation unaccounted for by other terms in the model and is modelled as $N(0,\sigma_{\varepsilon }^{2})$.

The Unadjusted model had no adjustments for county-level characteristics and quantifies the observed association of county-level death rates with their PM_2.5_ concentrations. The Covariate model adjusted for nine time-varying county-level covariates *X*_*i−county−year*_ (*i* = 1…9) (per capita income; percentage of population whose family income is below the poverty threshold, who are of Black or African American race, who have graduated from high school, who live in urban areas, and who are unemployed; a proxy for cumulative smoking; and mean temperature and relative humidity) with associated covariate-specific slopes, *θ*_*i*_. This model is analogous to the approach used by cohort studies, which typically adjust for baseline characteristics.

The Covariate-and-county model largely removed systematic variations in death rates and PM_2.5_ concentration across counties through the use of county-specific random intercepts (*α*_*county*_), hence implicitly adjusted for unobserved characteristics that systematically lead to higher or lower pollution and mortality in each county. In this model, the mortality effects of air pollution are mainly inferred through differential changes over time in death rates and PM_2.5_ concentrations across counties, similar to the difference-in-difference model used in econometric analyses [1,25]. The Restrictive model included both county-specific random intercepts and random slopes (*β*_*county*_), hence also implicitly adjusted for (linear) differential change in death rates and PM_2.5_ concentrations across counties and relates changes in PM_2.5_ concentration to changes in death rates after removing linear trends in mortality and pollution. By design, this model leaves limited spatial or temporal variation in death rates and estimates association with PM_2.5_ based only on the mortality residual not explained by linear trends. We included the Restrictive model because not only might level of mortality in a county be affected by unobserved/unmeasured factors, but so might its trend. All county-specific random intercepts and slopes were modelled using a conditional autoregressive (CAR) structure that (empirically) allows for death rates to be more similar across neighbouring counties than those that are further away.

We fitted the models using integrated nested Laplace approximation (INLA), using the R-INLA software, which offers orders of magnitude of computational efficiency improvement in Bayesian inference compared to traditional MCMC for latent Gaussian models. As in previous work [37], weakly informative hyperpriors of logGamma(1, 0.001) were specified on the logarithm of the precisions of the random effects so that the parameter estimates were data driven. Computer code for the models is available through http://www.globalenvhealth.org/.

Each model was applied to county-level cardiorespiratory death rates separately by sex and age group (5-year age groups from birth to 85 years and 85 years and older) because death rates vary by age group and sex, as might their associations with air pollution [38]. From each model, we estimated age-specific proportional increases in death rates (i.e., rate ratios) for each 1 μg/m^3^ of PM_2.5_ (the rate ratios are the exponential of the coefficients of PM_2.5_). We used the rate ratios from these models to calculate how many cardiorespiratory deaths in each five-year age group would be averted if PM_2.5_ concentration in each county were the same as the lowest observed concentration (2.8 μg/m^3^, in Apache, Arizona). This value is similar to the 2.5–3.5 μg/m^3^ concentration used by the US EPA as a policy-relevant background level [39]. We conducted a similar analysis to calculate the number of averted deaths and life expectancy gain as a result of PM_2.5_ reductions achieved from 1999 to 2015 by calculating the difference in the actual number of deaths in 1999 and those expected if PM_2.5_ concentration in each county had been at its 2015 level. In all scenarios, national estimates were obtained by summing the number of averted deaths in each age group across counties.

We used a lifetable to convert the estimated reduction in death rates that would be expected from lower concentrations to gain in life expectancy [32,40] at the county as well as national level. Specifically, we used the observed age-specific death rates to calculate life expectancy for each county or merged county unit, using standard lifetable techniques [40]. We calculated life expectancy under the counterfactual scenario of alternative PM_2.5_ concentration by subtracting the number of deaths from cardiorespiratory diseases in each age group if PM_2.5_ concentrations were at their alternative level from the observed number of deaths [32]. The difference between the observed and counterfactual life expectancy measures the contribution of PM_2.5_ pollution to life expectancy loss. In calculating life expectancies, we used the Kannisto–Thatcher method to expand the terminal (85 years and older) age group of the life table [41].

We report national and county results using the Covariate-and-county model as our main results because it fully adjusts for county characteristics through inclusion of covariates and random intercepts without being overly restrictive. To understand the impact of adjustments on our results, we compare national results from the Covariate-and-county model with those of the other three models.

Finally, we tested whether, after accounting for differences in PM_2.5_ concentration, life expectancy loss depends on county sociodemographic characteristics including per capita income, percentage of population whose family income is below the poverty threshold, percentage who are of Black or African American race, and percentage who have graduated from high school. For this purpose, we used a regression with life expectancy loss in 2015 estimated from the Covariate-and-county model as the dependent variable and PM_2.5_ concentration exceeding observed minimum of 2.8 μg/m^3^ and quintiles of each sociodemographic characteristic as independent variables. We report the difference between the first and fifth quintiles in Results.

## Results

In 2015, mean annual population-weighted PM_2.5_ concentrations in US counties ranged from 2.8 μg/m^3^ (Apache county, Arizona) to 13.2 μg/m^3^ (Tulare county, California) (Fig 1). Concentrations were below the current national ambient air quality standard for particle pollution of 12 μg/m^3^ in every county except 4 counties. In 1999, 59% of the 1,339 merged county units used in our analysis had concentrations greater than this level, with concentrations as high as 22.1 μg/m^3^ (Fresno county, California). Nationally, population-weighted average PM_2.5_ concentrations in the contiguous USA declined from 13.6 μg/m^3^ in 1999 to 8.0 μg/m^3^ in 2015. Pollution reduction was largest in the most polluted areas, such as southern California and some southern states (Alabama and Georgia), where PM_2.5_ declined by >10 μg/m^3^ from 1999 to 2015 (Fig 1).

**Fig 2:**
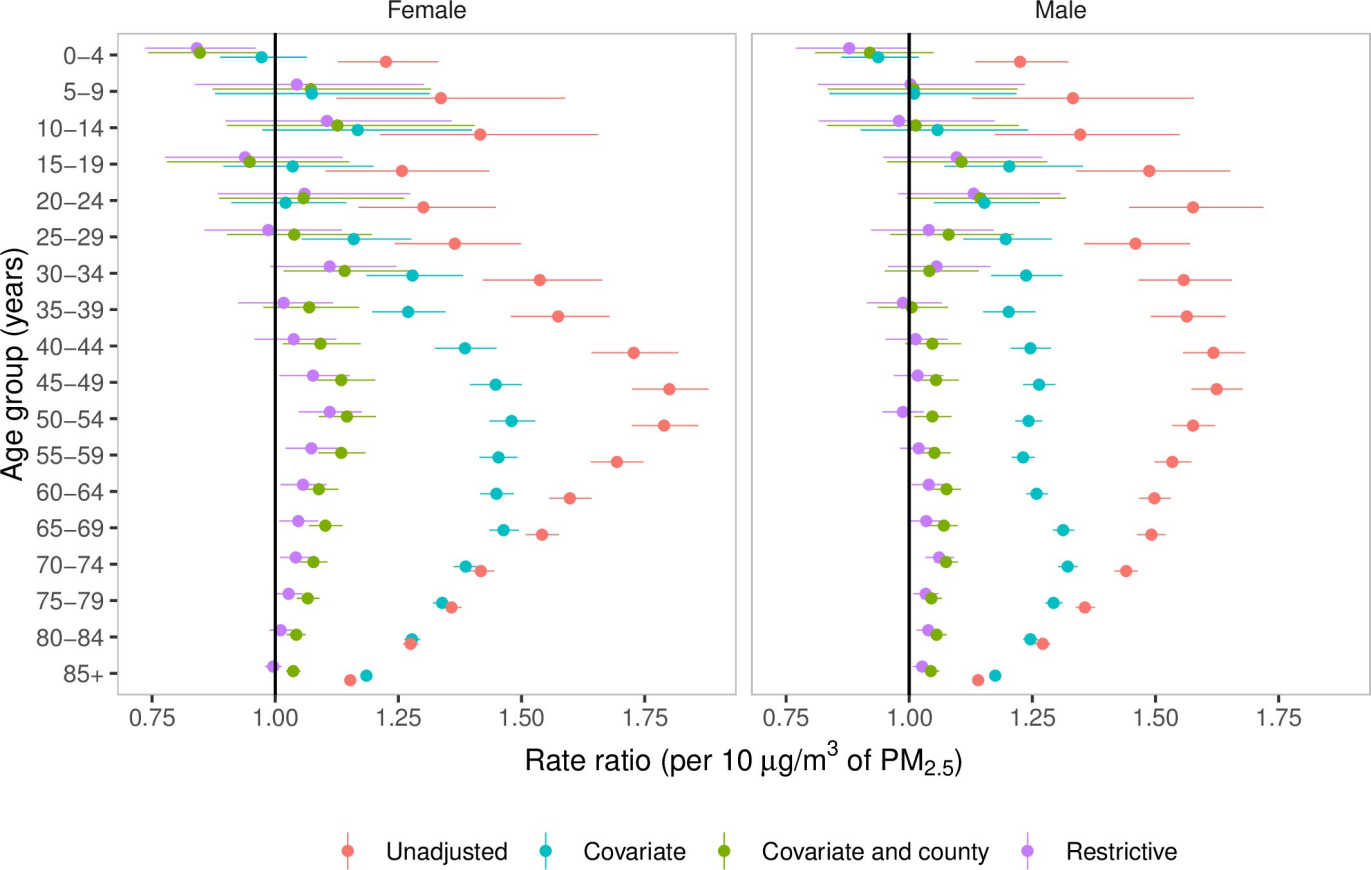
Sex- and age-group–specific rate ratios per 10 μg/m3 of PM_2.5_ for cardiorespiratory deaths. The rate ratios were estimated from four different Bayesian spatiotemporal models. PM_2.5_, fine particulate matter.

PM_2.5_ pollution in excess of the lowest observed concentration of 2.8 μg/m^3^ was associated with higher death rates from cardiorespiratory diseases (Fig 2). The rate ratios were largest in middle-aged adults, declining to smaller values in older ages as also seen for other cardiovascular risk factors [33,42]. Our main results, from the Covariate-and-county model—which adjusted for observed county characteristics, as well as for unobserved county characteristics through the use of county-specific random intercepts—were 15,612 (95% credible interval 13,248–17,945) deaths from cardiorespiratory diseases in females (2.8% of all female cardiorespiratory deaths) and 14,757 (12,617–16,919) deaths in males (2.7% of all male cardiorespiratory deaths). These deaths were equivalent to life expectancy losses of 0.15 years (0.13–0.17) in females and 0.13 years (0.11–0.15) in males, respectively.

**Fig 3:**
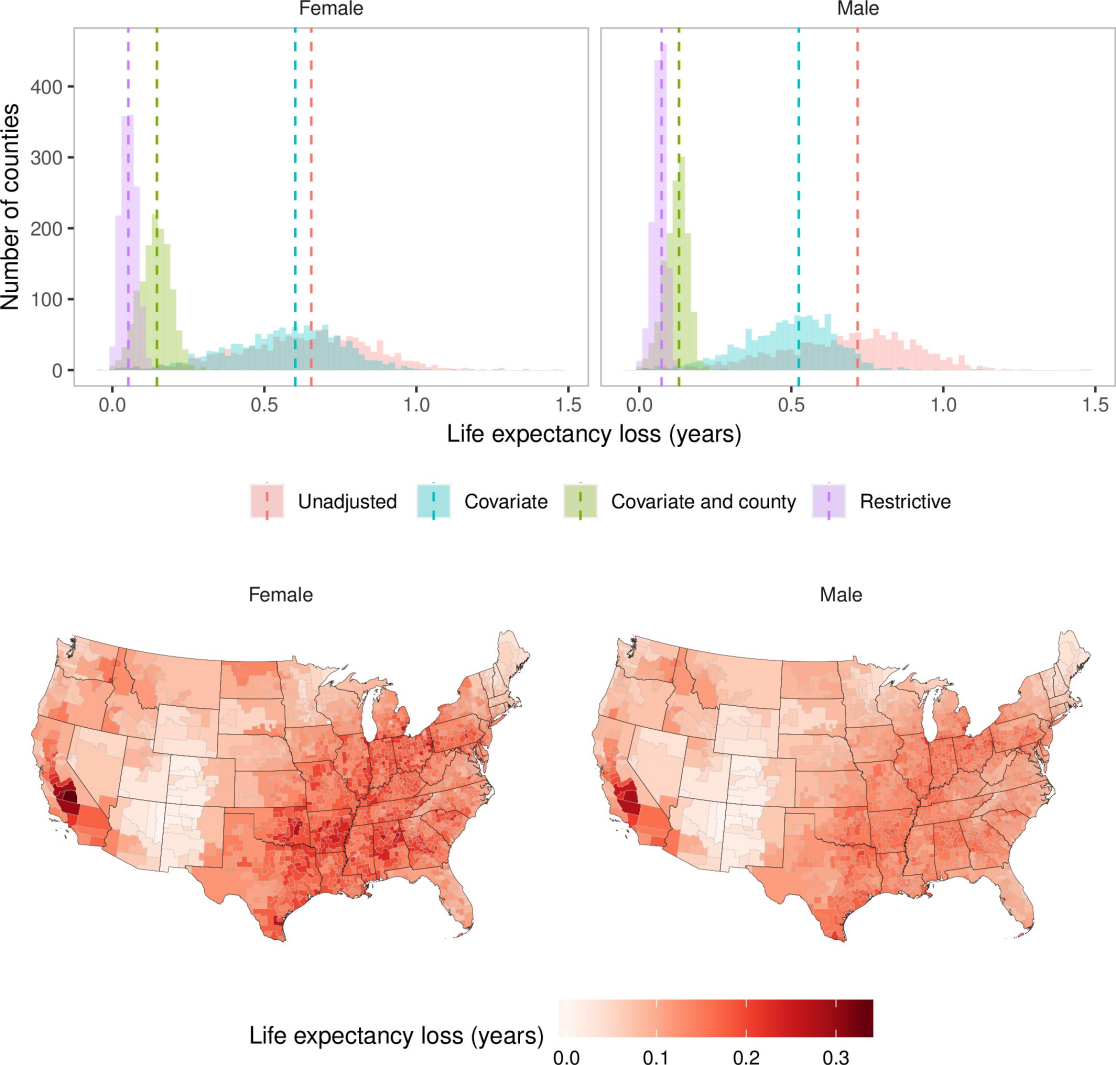
Life expectancy loss in 2015 from PM_2.5_ exceeding the observed minimum of 2.8 μg/m^3^. (A) Distribution of county-level life expectancy losses estimated from the four models (histograms) and the life expectancy losses at the national level estimated from the four models (dotted lines). (B) Life expectancy losses by county, estimated from the Covariate-and-county model. PM_2.5_, fine particulate matter.

The national results from the other three models show the impacts of varying degree of adjustment. In the Unadjusted model, which did not adjust for any county-level determinants of mortality, PM_2.5_ pollution in excess of the lowest observed concentration was associated with an estimated 67,053 (65,658–68,492) deaths from cardiorespiratory diseases (12.1% of all cardiorespiratory deaths) in females and 74,614 (73,330–75,913) deaths (13.6% of all cardiorespiratory deaths) in males. These deaths were equivalent to 0.66 (0.64–0.67) years lower life expectancy for females and 0.72 (0.71–0.73) years for males (Fig 3A). In the Covariate model, which adjusted for observed county characteristics, the number of deaths attributable to PM_2.5_ pollution were lowered to 64,578 (63,126–65,972) and 57,763 (56,529–59,023) for females and males, respectively, and the life expectancy loss to 0.60 (0.59–0.61) and 0.53 (0.51–0.54) years. Even the estimates from the Restrictive model showed 4,767 (1,739–7,715) deaths in females and 8,825 (6,169–11,491) deaths in males from PM_2.5_ pollution, equivalent to life expectancy losses of 0.05 (0.03–0.08) years and 0.07 (0.05–0.10) years, respectively.

County-level life expectancy loss in the main model, namely the Covariate-and-county model, ranged from <0.05 years in lower-pollution midwestern and Rocky Mountain counties that fall in states such as New Mexico, Colorado, and Arizona to >0.3 years in and around Los Angeles and in high-pollution and high-mortality counties in some southern states, such as Arkansas, Oklahoma, and Alabama (Fig 3B). Counties experiencing a loss of 0.2 years or more in female life expectancy accounted for 15.5% of the female population, and those experiencing a loss of 0.2 years or more in male life expectancy accounted for 8.5% of the male population.

**Fig 4:**
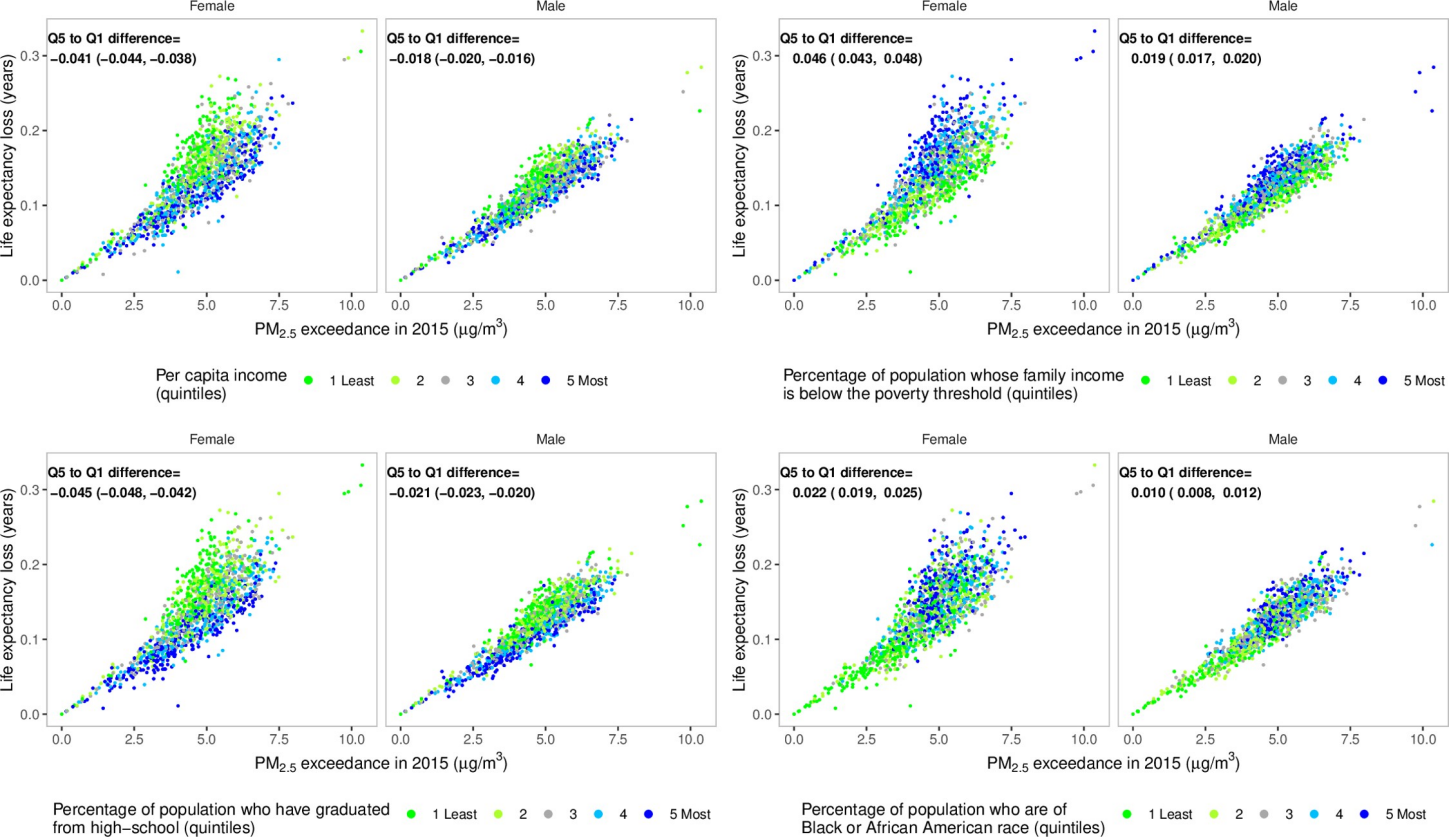
County-level life expectancy losses in 2015 from PM_2.5_ exceeding observed minimum of 2.8 μg/m^3^, estimated from the Covariate-and-county model, divided by quintiles of per capita income, proportion of population whose family income is below the poverty threshold, proportion of population who are of Black or African American race, or proportion of population who have graduated from high school. The estimated difference (with confidence interval) in life expectancy loss between quintile 5 and quintile 1, after accounting for PM2.5 concentration, is inset for each covariate and sex. The ranges in the five quintiles for per capita income are 17,400–24,900, 24,900–27,400, 27,400–30,100, 30,100–34,200, and 34,200–114,000 US dollars (adjusted for inflation with 2000 as the base year); for population whose family income is below the poverty threshold, 4%–11%, 11%–14%, 14%–16%, 16%–20%, and 20%–38%; for population who are of Black or African American race, 0%–2%, 2%–4%, 4%–8%, 8%–19%, and 19%–73%; for population who have graduated from high school, 46%–82%, 82%–86%, 86%–89%, 89%–91%, and 91%–97%. PM_2.5_, fine particulate matter; Q, quintile.

Life expectancy loss due to PM_2.5_ was larger in counties with lower income than in wealthier counties, counties with a higher proportion of population whose family income is below the poverty threshold, with a higher proportion of population who are of Black or African American race, or with a lower proportion of population who graduated from high school (Fig 4). This inequality in mortality burden occurs because lower-income counties, those with more poverty, with a greater proportion who are of Black or African American race, or with a lower proportion who have graduated high school tend to have higher baseline death rates at any pollution level because of conditions associated with these covariates and hence experience a larger absolute number of deaths as a result of air pollution. For example, after accounting for PM_2.5_ concentration, life expectancy loss in counties in the highest quintile of poverty compared to the lowest quintile is 0.046 years (confidence interval 0.043–0.048) lower in females and 0.019 years (0.017–0.020) lower in males.

**Fig 5:**
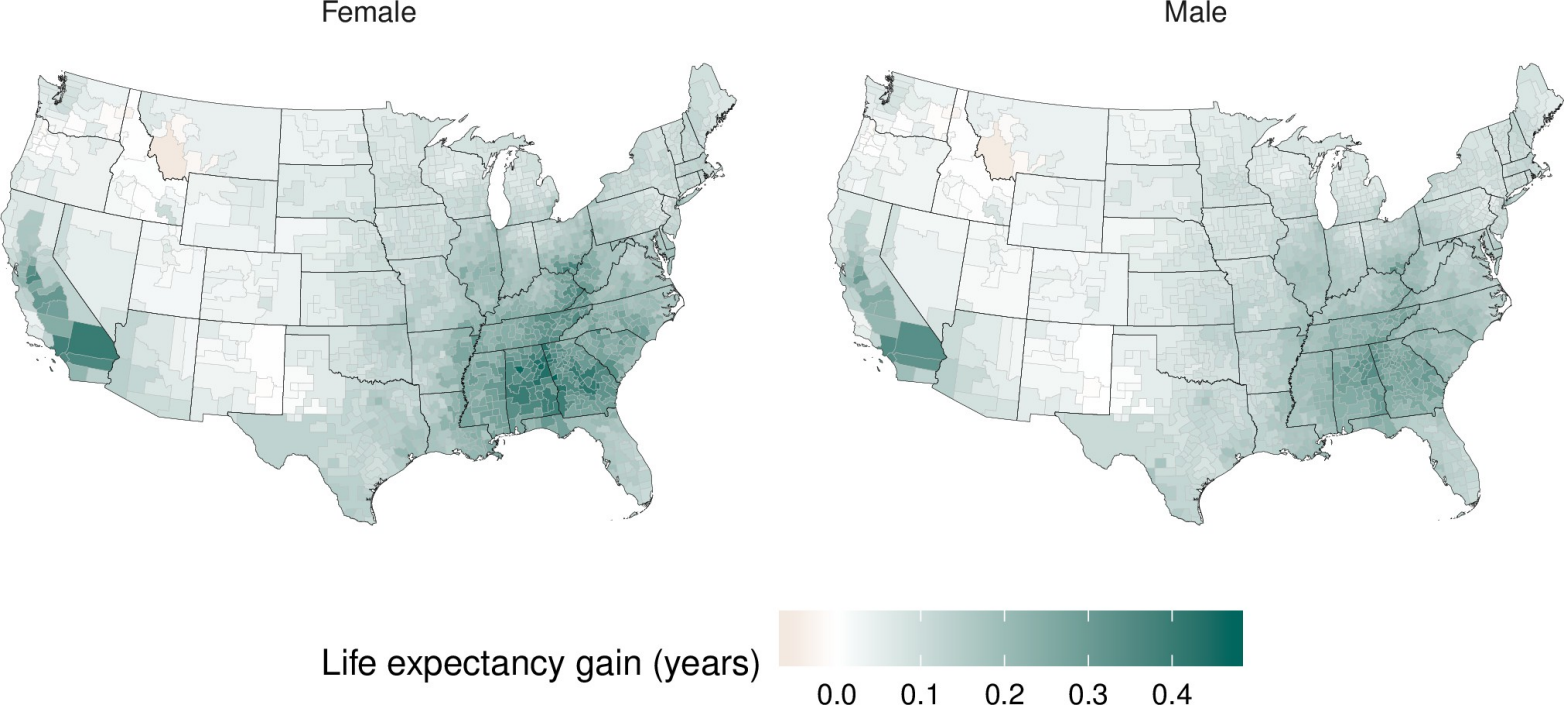
Contribution of PM_2.5_ reduction to life expectancy gains from 1999 to 2015, estimated using the Covariate-and-county model. PM_2.5_, fine particulate matter.

While current PM_2.5_ pollution is responsible for a significant mortality burden and loss of longevity, reductions in pollution since the late 1990s have benefited virtually the entire country, with the exception of 14 counties where PM_2.5_ increased slightly over this period (median increase of 0.2 μg/m^3^) (Fig 5). The national life expectancy gain as a result of these reductions was estimated from the Covariate-and-county model as 0.17 years (0.15–0.19) for females and 0.15 years (0.12–0.17) for males. For comparison, the overall observed increase in life expectancy in the contiguous USA from 1999 to 2015 was 2.0 and 2.5 years for females and males, respectively. The life expectancy gain estimated using the Covariate-and-county model surpassed 0.2 years in 393 counties (34.6% of population) for females and 227 counties (19.1% of population) for males. 117 counties (11.8% of population) for females and 27 counties (6.9% of population) for males, mostly in California and in some southern states (Alabama and Georgia), gained more than 0.3 years of life expectancy.

## Discussion

In this direct national analysis of mortality and life expectancy loss due to PM_2.5_ pollution, we estimate that current air pollution concentrations, which are relatively low by historical standards and mostly below the current national ambient air quality standard, are associated with a significant health burden after any reasonable control for other local determinants of mortality. The loss of longevity is larger in poorer counties, contributing to nationwide health inequalities. We also find that reductions in air pollution since 1999 have contributed to the longevity gains in the US population, with larger benefits especially in California and some southern states such as Alabama and Georgia, where PM_2.5_ declined more than the national average.

Two previous direct analyses of longevity loss due to air pollution in the USA focused on a subset of metro areas [1,25], largely before the current low concentrations were achieved, and hence are not directly comparable to our results. One study, using data from approximately 1980 to approximately 2000 from 211 counties in 51 metropolitan areas [1], found a life expectancy effect that was between the estimated effects of our Covariate-and-county and Covariate models. The other, using data from 2000 to 2007 in 545 counties, found a life expectancy effect similar to our Covariate-and-county model [25]. Neither study reported national or county-level life expectancy loss due to current pollution levels. Indirect estimate of life expectancy loss due to PM_2.5_ in the USA, with use of pooled hazard ratios from prospective cohort studies, is 0.38 years [24], which is midway between the estimates from our Covariate-and-county and Covariate models.

The main strength of our study is making direct estimates of the effects of PM_2.5_ pollution on death rates of the contemporary US population, nationally and for all counties or clusters of small counties. We further stratified our results by county sociodemographic characteristics, which reveals the role of air pollution on life expectancy inequalities. We present results using a range of model specifications, which shows how different adjustments for time-varying county-level determinants of mortality influence the estimated effect of air pollution. Finally, and importantly, the data used in our analysis are publicly available, as is the computer code for our statistical models, making our analysis fully transparent and replicable by others. Our analysis also meets the recent requirement of being based on publicly available data sets [16,43].

The main limitation of our study, similar to other observational studies, is that it is not guaranteed for the observed associations to be causal. We did not have annual county-level data on other important determinants of mortality, such as healthcare access and quality and diet. These factors are nonetheless implicitly adjusted for in our Covariate-and-county model. In particular, air pollution is a mixture of different pollutants. Of these, PM_2.5_ pollution has been consistently, independently, and coherently related to various diseases affected by air pollution [4,5,44,45]. Nonetheless, other pollutants, which may be partially correlated with PM_2.5_, may also have an effect. For example, the correlation coefficient between PM_2.5_ and ozone was 0.55 and between PM_2.5_ and nitrogen dioxide (NO_2_) 0.50 across all county-years in our analysis. Adjustment for ozone in the Covariate-and-county model led to slightly larger rate ratios for PM_2.5_, while adjustment for NO_2_ dampened the rate ratios, as also seen in an analysis of the Canadian Census Health and Environment Cohort [38]. However, according to the US EPA Integrated Science Assessment, current evidence is considered suggestive but not sufficient for a causal association between NO_2_ and cardiovascular mortality [46]; for ozone, the evidence was evaluated as suggestive for total mortality but as limited for cardiovascular mortality [47]. As described in the analysis of the Canadian Census Health and Environment Cohort, the impacts of these adjustments may be due to collinearity. Alternatively, it may be the case that some of the spatial variation in particle pollution that was not captured by the integrated geographic regression model is represented by ozone (typically aged or secondary, regional particle pollution) and/or NO_2_ (fresh and local particle pollution) [38], even if neither is directly related to cardiovascular mortality. For these reasons, ‘the results of the multi-pollutant models should be interpreted with caution’ [38]. Because the hazardous effects of smoking depend not only on current cigarette use but also on past smoking behaviour, including age at smoking initiation, duration of smoking, and number of cigarettes smoked per day, we used lung cancer death rate as the proxy for the cumulative hazards of smoking in a population. Although lung cancer death rate is a robust proxy for the cumulative hazards of smoking [33,34], it is also affected by air pollution, albeit to a much smaller degree than smoking, and hence its use to control for smoking leads to somewhat conservative estimates of the effect of air pollution. Finally, the modelled PM_2.5_ concentrations may have some error, which typically lowers the estimated effect of air pollution towards the null.

In summary, public health policy and regulatory efforts in the USA have spurred technological innovations and contributed to reduced air pollution. Although the health benefits of large pollution reductions in major metropolitan areas in the twentieth century [1] are widely acknowledged, there is policy resistance to actions that would further lower the concentration of pollutants such as PM_2.5_, most recently through requirements that constrain the evaluation of health effects of air pollution to publicly available data sets [16,43]. Our direct national analysis of the mortality burden of PM_2.5_ pollution, using publicly available data and with a range of analytical models, indicate that current PM_2.5_ pollution continues to contribute to mortality and loss of longevity in the USA, with larger harms in poorer counties. Our results suggest that further lowering PM_2.5_ pollution is likely to benefit the health of the entire US population, and lower health inequalities.

## Supporting information

S1 STROBE Checklist STROBE, Strengthening the reporting of observational studies in epidemiology. (DOC)

## Abbreviations

CAR: conditional autoregressive
EPA: Environmental Protection Agency
ICD: International Classification of Disease
INLA: integrated nested Laplace approximation
NCHS: National Center for Health Statistics
PM_2.5_: fine particulate matter
