# Supplementary material for: Particulate matter air pollution and national and county life expectancy loss in the USA: A spatiotemporal analysis
Source: PLoS Med. 2019 Jul 23;16(7):e1002856. doi: 10.1371/journal.pmed.1002856 (PMC6650052; doi:10.1371/journal.pmed.1002856)
Supplement: S1 STROBE Checklist — (DOC) [file pmed.1002856.s001.doc]

STROBE Statement—Checklist of items that should be included in reports of ***cross-sectional studies***

|  | Item No | Recommendation |
| --- | --- | --- |
| **Title and abstract** | 1 | (*a*) Indicate the study’s design with a commonly used term in the title or the abstract **Yes, in the title, spatio-temporal analysis.** |
|  |  | (*b*) Provide in the abstract an informative and balanced summary of what was done and what was found **Yes, in the abstract.** |
| Introduction | | |
| Background/rationale | 2 | Explain the scientific background and rationale for the investigation being reported **Yes, in the Introduction.** |
| Objectives | 3 | State specific objectives, including any prespecified hypotheses **Yes, in the final paragraph of the Introduction.** |
| Methods | | |
| Study design | 4 | Present key elements of study design early in the paper **Yes, in Methods/Study design.** |
| Setting | 5 | Describe the setting, locations, and relevant dates, including periods of recruitment, exposure, follow-up, and data collection **Yes, in Methods sections Study design, Data sources and Units of analysis.** |
| Participants | 6 | (*a*) Give the eligibility criteria, and the sources and methods of selection of participants **NA, we included all deaths in contiguous USA during the study period.** |
| Variables | 7 | Clearly define all outcomes, exposures, predictors, potential confounders, and effect modifiers. Give diagnostic criteria, if applicable **Yes, in Methods sections Study design and Data sources and Table 1.** |
| Data sources/ measurement | 8* | For each variable of interest, give sources of data and details of methods of assessment (measurement). Describe comparability of assessment methods if there is more than one group **Yes, in Methods/Data sources and Table 1.** |
| Bias | 9 | Describe any efforts to address potential sources of bias **Yes, we investigated the effects of over and under adjustment for potential confounders and spatiotemporal model terms by considering four separate models (see Table 2).** |
| Study size | 10 | Explain how the study size was arrived at **NA, see Methods/Study design, we included all deaths in contiguous USA during the study period.** |
| Quantitative variables | 11 | Explain how quantitative variables were handled in the analyses. If applicable, describe which groupings were chosen and why **NA** |
| Statistical methods | 12 | (*a*) Describe all statistical methods, including those used to control for confounding **Yes, in Methods/Statistical methods and Table 2** |
|  |  | (*b*) Describe any methods used to examine subgroups and interactions **NA** |
|  |  | (*c*) Explain how missing data were addressed **Yes, in Methods/Data sources, two potential confounders did not have complete time series and linear interpolation was used.** |
|  |  | (*d*) If applicable, describe analytical methods taking account of sampling strategy **NA** |
|  |  | (*e*) Describe any sensitivity analyses **Yes, see description of the four models in Methods/Statistical methods and Table 2.** |
| Results | | |
| Participants | 13* | (a) Report numbers of individuals at each stage of study—eg numbers potentially eligible, examined for eligibility, confirmed eligible, included in the study, completing follow-up, and analysed **Yes, in Methods/Data sources paragraph 1.** |
|  |  | (b) Give reasons for non-participation at each stage **NA** |
|  |  | (c) Consider use of a flow diagram **NA** |
| Descriptive data | 14* | (a) Give characteristics of study participants (eg demographic, clinical, social) and information on exposures and potential confounders **See summary statistics of counties in Table 1.** |
|  |  | (b) Indicate number of participants with missing data for each variable of interest **NA** |
| Outcome data | 15* | Report numbers of outcome events or summary measures **Yes, in Methods/Data sources paragraph 1.** |
| Main results | 16 | (*a*) Give unadjusted estimates and, if applicable, confounder-adjusted estimates and their precision (eg, 95% confidence interval). Make clear which confounders were adjusted for and why they were included **Yes, in Results.** |
|  |  | (*b*) Report category boundaries when continuous variables were categorized **Yes, Fig 4 caption.** |
|  |  | (*c*) If relevant, consider translating estimates of relative risk into absolute risk for a meaningful time period **Yes, in Results, rate ratios have been used to calculate life expectancy losses due to PM2.5.** |
| Other analyses | 17 | Report other analyses done—eg analyses of subgroups and interactions, and sensitivity analyses **Yes. Results of all 4 models are presented in paragraphs 1-3 of Results.** |
| Discussion | | |
| Key results | 18 | Summarise key results with reference to study objectives **Yes, in Discussion paragraph 1.** |
| Limitations | 19 | Discuss limitations of the study, taking into account sources of potential bias or imprecision. Discuss both direction and magnitude of any potential bias **Yes, in Discussion paragraph 3.** |
| Interpretation | 20 | Give a cautious overall interpretation of results considering objectives, limitations, multiplicity of analyses, results from similar studies, and other relevant evidence, **Yes, in Discussion paragraphs 1,2,4.** |
| Generalisability | 21 | Discuss the generalisability (external validity) of the study results **NA** |
| Other information | | |
| Funding | 22 | Give the source of funding and the role of the funders for the present study and, if applicable, for the original study on which the present article is based**. Yes, see Financial disclosure field. The paper has not been formally reviewed by EPA. The views expressed in this document are solely those of authors and do not necessarily reflect those of the Agency. EPA does not endorse any products or commercial services mentioned in this publication.** |

*Give information separately for exposed and unexposed groups.

**Note:** An Explanation and Elaboration article discusses each checklist item and gives methodological background and published examples of transparent reporting. The STROBE checklist is best used in conjunction with this article (freely available on the Web sites of PLoS Medicine at http://www.plosmedicine.org/, Annals of Internal Medicine at http://www.annals.org/, and Epidemiology at http://www.epidem.com/). Information on the STROBE Initiative is available at www.strobe-statement.org.
